# Effects of postoperative hand therapy in patients with Dupuytren’s disease: A prospective hyperspectral imaging study

**DOI:** 10.1007/s00132-025-04631-w

**Published:** 2025-03-20

**Authors:** F. M. Lorenz, E. Henning, C. Sicher, I. Langner

**Affiliations:** 1 Department of Hand Surgery, Center for Orthopaedics, Trauma Surgery and Rehabilitation Medicine, University Medicine Greifswald, Ferdinand-Sauerbruch-Straße, 17475 Greifswald, Germany; 2 Section Epidemiology of Health Care and Community Health, Institute for Community Medicine, University Medicine Greifswald, Greifswald, Germany; 3 Institute of Hygiene and Environmental Medicine, University Medicine Greifswald, Greifswald, Germany

**Keywords:** Dupuytren’s disease, Hand therapy, Hyperspectral imaging, Microperfusion, Dupuytren’sche Kontraktur, Handtherapie, Hyperspektrale Bildgebung, Mikrozirkulation

## Abstract

**Background:**

Hyperspectral imaging enables noninvasive evaluation of the microcirculation, which affects wound healing. In patients with Dupuytren’s disease the microcirculation should be improved in order to achieve good clinical results and reduce the risk of recurrence. The aim of the study was to evaluate the microcirculatory effectiveness of postoperative hand therapy in patients with Dupuytren’s disease after partial fasciectomy.

**Methods:**

In an inpatient hospital setting 35 patients with Dupuytren’s disease were investigated before and after partial fasciectomy. Standardized hand therapy was performed after surgery. Its effects on superficial and deep oxygenation, tissue hemoglobin index and tissue water index were assessed using hyperspectral imaging at 3 regions of interest (ROI) within the first 2 postoperative days. For image evaluation, three ROIs were placed manually within the palm, the fingertip of the affected digit (fourth or fifth digit) and a fingertip of a control digit (unaffected, second digit of the same hand) using the vendor’s software (Tivita^TM^ Tissue Suite, Diaspective Vision, Germany).

**Results:**

Superficial oxygenation increased 1 day after surgery and after hand therapy. The tissue water index decreased on the second postoperative day.

**Conclusion:**

Hyperspectral Imaging demonstrates that postoperative hand therapy effectively improves perfusion and oxygenation in the hands of patients with Dupuytren’s disease and additionally reduces edema.

## Abstract (de)

**Hintergrund:**

Die hyperspektrale Bildgebung ermöglicht eine nichtinvasive Beurteilung der Mikrozirkulation, die einen Einfluss auf die Wundheilung hat. Bei Patienten mit Morbus Dupuytren sollte die Mikrozirkulation verbessert werden, um ein gutes klinisches Ergebnis zu erzielen und das Risiko eines Rezidivs zu reduzieren. Das Ziel der Studie war es, den Einfluss der postoperativen Handtherapie auf die Mikrozirkulation bei Patienten mit Morbus Dupuytren nach partieller Fasziektomie zu beurteilen.

**Methoden:**

Insgesamt 35 stationär behandelte Patienten mit Morbus Dupuytren wurden vor und nach einer partiellen Fasziektomie untersucht. Eine standardisierte Handtherapie wurde nach der Operation durchgeführt. Ihre Effekte auf die oberflächliche und tiefe Sauerstoffversorgung, den Gewebshämoglobinindex und den Gewebswasserindex wurden mittels hyperspektraler Bildgebung in drei definierten Bereichen innerhalb der ersten zwei postoperativen Tage gemessen. Hierfür wurde händisch eine ROI in die Handfläche, in die Fingerspitze des betroffenen Fingers (vierter oder fünfter Finger) und im Bereich der Fingerspitze des Kontrollfingers (nichtbetroffener Zeigefinger der gleichen Hand) mit der Software des Herstellers eingelegt.

**Ergebnisse:**

Einen Tag nach der Operation führte die Handtherapie zu einer Erhöhung der oberflächlichen Sauerstoffversorgung. Am zweiten postoperativen Tag nahm der Gewebswasserindex ab.

**Schlussfolgerung:**

Die hyperspektrale Bildgebung zeigt, dass die postoperative Handtherapie die Durchblutung und Sauerstoffversorgung der Hand bei Patienten mit Dupuytren-Kontraktur effektiv verbessert und zusätzlich Ödeme reduziert.

## Introduction

Dupuytren’s disease (DD) is a benign fibroproliferative connective tissue disease of the palmar aponeurosis. The prevalence of DD varies from 0.6% to 31.6% [1] and partial fasciectomy is one of the most common elective operations in hand surgery [2]. The disease is characterized by proliferation of fibroblasts alongside differentiation to myofibroblasts, the expansion of collagen type III and an increasing fibrotic remodelling of palmar and digital aponeuroses and the surrounding soft tissues including the skin [3].

The exact pathophysiology of DD is still unknown, the observed accumulation of immune cells and proinflammatory cytokines and occluded capillaries give reason to believe that the proliferation of myofibroblasts is part of an immunologically triggered microvascular damage similar to acute wounds [3, 4]. Knuckle pads may develop on the dorsal side of the proximal interphalangeal joint but palmar thickening of the aponeurosis in the form of nodules, cords, and later nodule-cord units mainly occurs, which in turn leads to progressive shortening of the aponeurosis [5, 6]. Further involvement of joint capsules fixates the flexion contractures of the affected digits (usually 4th and/or 5th digit) and thus to a functional limitation.

There are a several treatment options for primary and recurrent DD: radiotherapy and multiple minimally invasive treatment options, such as percutaneous needle fasciotomy, corticoid steroid injection or *Clostridium histolyticum* collagenase injection [7–9]. Nevertheless, surgery remains the main treatment option with partial fasciectomy being the gold standard [10]. Indications for partial fasciectomy were a flexion contracture with positive tabletop test, a coexisting trigger finger or pain or skin irritation caused by nodules. In addition to patient instructions for cooling and elevation [8] during the postoperative period, splinting and hand therapy (HT) are recommended.

Complication rates are variably reported, with wound healing disorders, pain, dysesthesia and flare reaction being the most common [11]. Recurrence is commonly observed in DD and occurs in 9–27% of patients [12, 13]. Dupuytren’s disease is a systematic and chronic disease and the same factors which seems to be etiological for its primary formation are considered to trigger recurrences, i.e., the alteration of microperfusion; however, microperfusion can be improved by local exercise therapy [14].

Hyperspectral imaging (HSI) enables the noninvasive qualitative and quantitative assessment of perfusion parameters and the water content of tissue in vivo, thus enabling the understanding of complex physiological processes of acute and chronic wounds [15–17].

In this study, we hypothesized that HT has an impact on tissue microperfusion and water content in patients with DD after partial fasciectomy.

## Material and methods

### Study population

During the study period between March 2018 and February 2020 all patients with palmar DD who underwent partial fasciectomy were prospectively included. Additional inclusion criteria were age of ≥ 18 years and ≤ 90 years. Exclusion criteria were general contraindications for surgery. All participants gave written informed consent and signed the privacy policy before participation.

Preoperatively, all patients were classified according to the criteria of Tubiana et al. concerning the flexion contracture in each ray [18]. We assessed cardiovascular risk factors and factors impairing wound healing, such as nicotine consumption, body mass index (BMI), diabetes mellitus, peripheral arterial occlusive disease and arterial hypertension [19–22]. Important disease-related factors such as recurrence of DD and complications during the first operation (lesions of the neurovascular bundle), positive family history of DD and operating times were recorded. Recurrent DD was defined as newly developed passive extension deficit of more than 20° and palpable cords for at least one of the treated joints more than 3 months after the first treatment [23].

### Surgical technique

Surgery was performed by a single board-certified hand surgeon. Partial fasciectomy was undertaken in a standardized way via mini-Bruner incisions; however, individual factors such as the overall extent of the contracture or the presence and quantity of nodules, cords and nodule-cord units, led to an individualization of the operation at the discretion of the surgeon. Due to Y‑V plasty no skin graft was required.

The fascia material obtained during surgery was examined histologically and classified according to the pathological Luck’s stages I–III (proliferative stage, involutional stage, residual stage) [6].

All patients received a 6F drainage and a palmar compression splint with approximately 40 mm Hg, monitored by a pressure sensor placed on the palm of the hand, extending from the distal forearm to the fingertips 2–5 fixated by an elastic bandage and wadding in neutral position of all joints.

### Hand therapy

Standardized HT was performed by a specialized hand therapist, twice on the first and second postoperative days for 20 min each in the morning and afternoon with about 6 h between the two exercises. After splint removal every patient received manual drainage to reduce edema of the digits and the palm, from proximal to distal in a pain-adapted manner, followed by an incipient wound treatment with circling massage movements and applying pressure around the wound. Passive stretches and passive movements of all interphalangeal and metacarpophalangeal joints were started in functional positions and ended in near full flexion and extension. Subsequently patients had to perform slow active movement to near full flexion and extension.

After HT the compression splint was applied in an identical scheme as with the intraoperative splinting.

### Hyperspectral imaging

For hyperspectral imaging, the TIVITA^TM^ tissue camera (Diaspective Vision, Am Salzhaff-Pepelow, Germany) was used. This system can record a hyperspectral image in a fast (3–15 s for image acquisition) and contact-free manner (50 cm distance). Using broadband light-emitting diodes, the HSI camera is able to capture an image with an in-plane resolution of 640 × 480 pixels. Spectral information for each pixel is recorded in a range of 500–1000 nm, creating a virtual three-dimensional data cube (two spatial and one spectral dimension). At a spectral resolution of 5 nm, 100 spectral channels for each pixel are obtained for further analysis. The vendor’s software calculates the ratio of oxygenated hemoglobin, the total hemoglobin concentration and the corresponding relative oxygenation and its distribution in superficial and subcutaneous areas [15, 24]. From the acquired data, the following physiological parameters of microcirculation were recorded: StO2 (relative oxygen saturation in superficial tissue layers in percentage; penetration depth approximately 1 mm), near infrared perfusion index NIR (index value of the relative oxygen saturation in deep tissue layers; penetration depth up to 6 mm), tissue hemoglobin index THI (index value of the relative quantity of hemoglobin; penetration depth approximately 1 mm) and tissue water index TWI (index value of the relative quantity of water in the tissue: penetration depth up to 6 mm) [25].

**Fig. 1:**
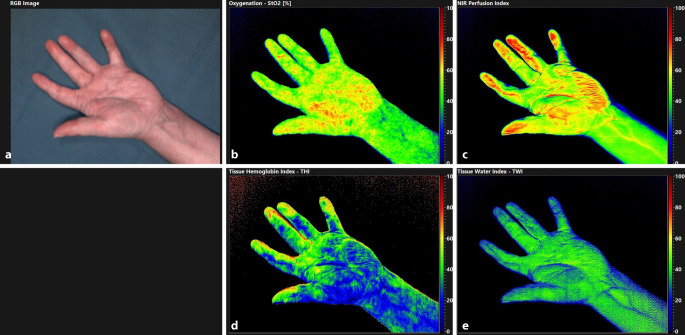
Hyperspectral Imaging exemplary for one patient. **a** color-coded image which allows the positioning of the ROIs, **b** StO_2_, **c** NIR, **d** THI, **e** TWI. StO_2_ in %, THI, TWI and NIR as index values from 0 to 100; *NIR* near infrared perfusion index, *StO2* relative oxygen saturation, *THI* tissue hemoglobin index, *TWI* tissue water index

Image acquisition was performed once prior to surgery in the outpatient clinic at room temperature (18 °C) and on the first (OP + 1) and second (OP + 2) postoperative days immediately before and after the first HT session of each day. Postoperative imaging acquisition was performed in a dedicated examination room on the ward again at room temperature. The first session was used to keep the effect of possible influencing factors (i.e., smoking, stress factors in the hospital) of the individual patient’s daily routine as low as possible. For image evaluation, three regions of interest (ROI) were placed manually within the palm, the fingertip of the affected digit (fourth or fifth digit) and a fingertip of a control digit (unaffected, second digit of the same hand) using the vendor’s software (see Fig. 1). The ROIs are defined by the HSI system as circular areas, which were individually adapted to the patient and for which the maximum possible area of the fingertips or palm was included.

### Statistical analysis

Data were analyzed using IBM SPSS Statistics (IBM Corp. Released 2023. IBM SPSS Statistics for Windows, Version 29.0.2.0 Armonk, NY, USA). Continuous variables are presented as mean with standard deviation (SD) or as median with range. Categorical variables are displayed as numbers and percentages. Continuous and normally distributed data in categorized groups were compared through the Lavene test for variance homogeneity and t‑test for independent samples. Paired t‑tests were used for data with interval samples. Using Fisher’s exact test categorical variables were compared. *P*-values ≤ 0.05 were considered as statistically significant.

The study was approved by the local ethics committee (BB 041/18a) and was performed according to the principles of the World Medical Association (WMA) Declaration of Helsinki.

## Results

### Patient population

During the study period 35 patients were included and 6 of them were female. All patients are northwestern Europeans with a similar fair skin type. The mean age of the patient group was 63.5 years (SD ±8.5 years). Of the patients 16 had a recurrence of DD, who in turn had no reported lesions of the neurovascular bundle from the previous operation. The DD was confirmed histologically in all patients: a proliferative stage was seen in 16, involutional stage in 13 and residual stage in 5 patients, with 1 result missing. There were no significant histological differences observed in primary and recurrent DD and no scar tissue was seen in nodules and cords of recurrent DD.

Further demographic details are provided in Table 1.

**Table 1:** Demographic and clinical data of all patients: primary DD and recurrent DD group

Characteristics	All patients (*n* = 35)	Primary DD (*n* = 19)	Recurrent DD (*n* = 16)
*Male, n*	29	17	12
*Female, n*	6	2	4
*Age* (years; mean ± SD)	63.5 ± 8.5	62.9 ± 6.2	64.2 ± 10.8
(median, range)	(62, 47–80)	(61, 54–75)	(63, 47–80)
*BMI* (kg/m^2^; mean ± SD)	27.6 ± 5.2	27.9 ± 6.0	27.1 ± 4.1
(median, range)	(26.5, 19.4–46.1)	(26.8, 19.4–46.1)	(26.0, 21.2–37.1)
**Risk factors**			
*Diabetes mellitus, n*	9	6	3
*Nicotine, n*	9	4	5
*PAOD, n*	5	2	3
*AHT, n*	16	8	8
**Disease related factors**			
*Positive family history, n*	15	9	6
*Operating time* (min; mean ± SD)	88.4 ± 0.8	74.67 ± 23.8	104.3 ± 31.0
Mean (median, range)	(86, 41–164)	(78, 41–113)	(90, 65–164)
**Tubiana stage, *n***			
*I*	9	7	2
*II*	19	11	8
*III*	5	0	5
*IV*	2	1	1
**Luck’s histology stage, *n***			
*Missing*	1	1	0
*Proliferative stage*	16	11	5
*Involutional stage*	13	6	7
*Residual stage*	5	1	4

*BMI* body mass index, *PAOD* peripheral arterial occlusive disease, *AHT* arterial hypertension

In addition, we compared patients operated on with primary DD (*n* = 19) and with recurrent DD (*n* = 16). The primary group had a lower stage in the Tubiana classification (*p* = 0.026). The mean operating time of 75 min (SD 24) in the primary group was also significantly less than 104 min (SD 31) in the recurrence group (*p* = 0.007). In patients with recurrent DD, the last surgical treatment was a mean of 7.3 years (SD 4.6) before the current operation (min. 1 year; max. 16 years). There were no other significant differences in any of the assessed baseline characteristics.

### Hyperspectral imaging

Preoperative HSI was performed on average 8.7 days (SD 22.1) before surgery in all patients.

In Table 2, all patient’s preoperative values are listed below each other. Comparing individual ROI with one-another, the highest StO2 value is found in the control digit (71.81%), followed by the palm (63.25%, *p* < 0.001) and the affected digit (61.92%, *p* < 0.001). THI was also highest in the control digit (58.59), followed by the affected digit (53.23, *p* < 0.001) and then by the palm (43.43, *p* < 0.001). TWI was lowest on the control digit, followed by the palm (50.93, *p* < 0.001) and the affected digit (48.92, *p* < 0.001). NIR was again highest at the control digit (61.82), with the palm (56.75, *p* < 0.001) and the affected digit (55.62, *p* < 0.001) being lower.

**Table 2:** Mean HSI values (StO2, THI, TWI, NIR) preoperatively, before and after HT for the patient group

ROI	Value	preOP	OP + 1				OP + 2			
			preHT	postHT	Change	*p*-value	preHT	postHT	Change	*p*-value
Palm	StO_2_	63.25	**62.20** **±** **3.75**	**63.63** **±** **3.41**	**2.30%**	**0.004**	62.50 ± 4.91	62.79 ± 4.95	0.47%	0.469
	THI	43.43	**48.23** **±** **9.04**	**49.49** **±** **9.58**	**2.61%**	**0.011**	48.35 ± 10.28	48.12 ± 9.63	−0.49%	0.628
	TWI	50.93	**45.89** **±** **3.87**	**44.20** **±** **4.21**	**−3.67%**	**<** **0.001**	**45.50** **±** **4.15**	**43.74** **±** **4.56**	**−3.88%**	**<** **0.001**
	NIR	56.75	**60.03** **±** **4.55**	**58.80** **±** **4.35**	**−2.05%**	**0.003**	60.56 ± 4.45	59.97 ± 4.42	−1.12%	0.062
AD	StO_2_	61.92	**45.06** **±** **7.55**	**46.14** **±** **7.51**	**2.41%**	**0.008**	**44.24** **±** **8.01**	**44.94** **±** **8.53**	1.60%	0.113
	THI	53.23	**73.83** **±** **10.86**	**76.97** **±** **11.03**	**4.26%**	**<** **0.001**	**75.21** **±** **16.23**	**76.79** **±** **11.04**	2.11%	0.225
	TWI	48.92	32.49 ± 8.61	31.23 ± 6.78	−3.87%	0.090	**31.74** **±** **6.88**	**29.82** **±** **7.92**	**−6.02%**	**0.001**
	NIR	55.62	42.69 ± 10.30	42.69 ± 10.23	0.00%	1.000	40.59 ± 10.33	40.91 ± 11.16	0.80%	0.664
CD	StO_2_	71.81	**68.00** **±** **10.42**	**70.56** **±** **9.70**	**3.76%**	**0.012**	68.03 ± 10.42	68.62 ± 10.69	0.86%	0.432
	THI	58.59	65.74 ± 13.25	66.53 ± 13.77	1.21%	0.333	62.26 ± 11.07	61.74 ± 10.73	−0.85%	0.487
	TWI	43.30	45.71 ± 4.98	45.09 ± 5.43	−1.35%	0.325	**46.47** **±** **5.13**	**45.48** **±** **4.77**	**−2.53%**	**0.008**
	NIR	61.82	63.62 ± 7.33	62.74 ± 6.98	−1.39%	0.326	62.50 ± 6.50	61.71 ± 9.02	−1.27%	0.429

HSI values (mean significant changes *p* < 0.05) highlighted with bold font; StO2 in %; THI, TWI and NIR as index values from 0 to 100

*ROI* region of interest, *AD* affected digit, *CD* control digit, *HT* hand therapy, *HSI* hyperspectral imaging, *NIR*  near infrared perfusion index, *preOP* HSI values before surgery, *OP* *+* *1* first day after surgery, *OP* *+* *2* second day after surgery, *preHT* before HT, *postHT* after HT, *StO2* relative oxygen saturation, *THI* tissue hemoglobin index, *TWI* tissue water index

Recurrent and primary DD did not show any significant differences in preoperative measured StO2, THI and TWI, with only the preoperative NIR being higher in the primary group in all three ROIs (palm *p* = 0.018; affected digit *p* = 0.011; control digit *p* = 0.005) and THI in the affected digit being lower (*p* = 0.035) in the primary group.

When comparing preoperative measurements with those obtained after surgery (at OP + 1 before HT) StO2 decreased in the affected digit (*p* < 0.001). The TWI decreased in the affected digit (*p* < 0.001) and the palm (*p* < 0.001) and increased in the control digit (*p* = 0.044). The THI increased in all three ROIs (palm *p* = 0.003; affected digit *p* < 0.001; control digit *p* = 0.001). The NIR increased in the palm (*p* = 0.042) and decreased in the affected digit (*p* < 0.001).

**Fig. 2:**
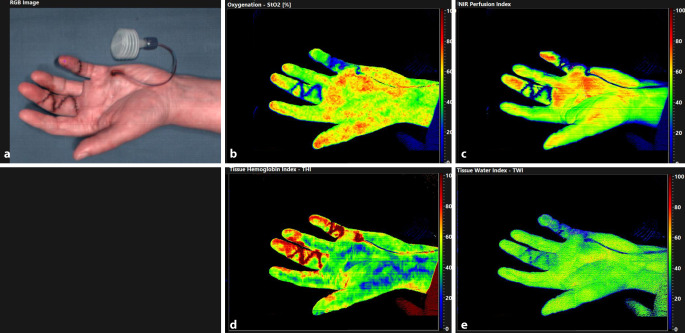
Hyperspectral Imaging of the same patient as in Fig. 1 on day OP + 2. **a** color-coded image which enables the positioning of the ROI; **b** StO_2_; **c** NIR; **d** THI; **e** TWI. StO_2_ in %; THI, TWI and NIR as index values from 0 to 100; *NIR* near infrared perfusion index, *StO2* relative oxygen saturation, *THI* tissue hemoglobin index, *TWI* tissue water index

The first two columns of Table 2 show measurements before and after HT subdivided according to OP + 1 and OP + 2. The HSI measurements at OP + 1 showed an increase in StO2 in all three ROI (palm *p* = 0.004; affected digit *p* = 0.008; control digit *p* = 0.012). The THI increased in the palm (*p* = 0.011) and the affected digit (*p* < 0.001). The TWI (*p* < 0.001) and NIR (*p* = 0.003) decreased exclusively in the palm. At OP + 2, exclusively TWI values decreased at all three ROI after HT (palm *p* < 0.001; affected digit *p* = 0.001; control digit *p* = 0.008; see Fig. 2).

**Fig. 3:**
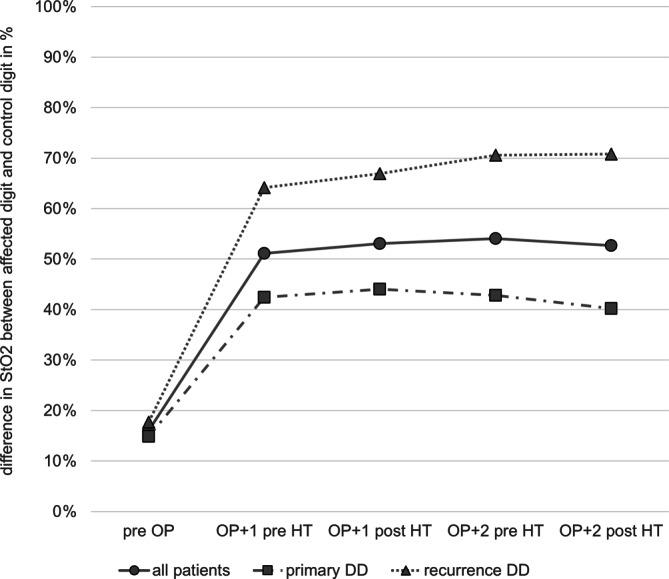
Mean StO_2_ differences between affected digit and control digit in percentage. *preOP* 1 day before operation, *preHT* before HT, *postHT* after HT, *OP* *+* *1* first day after surgery, *OP* *+* *2* second day after surgery. *Circle* all patients, *square* patients with primary DD, *triangle* patients with recurrent DD

The StO2, TWI and NIR differed between the affected digit and the control digit. This discrepancy is visualized in Fig. 3 and was more pronounced with increasing treatment duration. The StO2 at the control digit compared to the affected digit were 16% higher preoperatively, 51% higher before and 53% higher after HT at OP + 1, 54% higher before and 53% higher after HT at OP + 2. The TWI at the control digit versus the affected digit was 12% lower preoperatively, 42% higher before and 46% higher after HT at OP + 1, 48% higher before and 56% higher after HT at OP + 2. The NIR at the control digit was 11% higher preoperatively, 49% higher before and 47% higher after HT at OP + 1, 54% higher before and 50% higher after HT at OP + 2.

## Discussion

Partial fasciectomy remains the gold standard in the treament of DD followed by compression, splinting and HT in the postoperative period to support wound healing and improve hand function [10, 26]. Recommendations for HT concerning duration, timing, intensity and technique are mostly based on experience [8]. Scientific evidence on its effectiveness and physiological mechanism is absent.

The HSI enables the noninvasive qualitative and quantitative evaluation of microcirculation parameters and tissue water content near to real time and provides a sound and direct feedback of treatment effects [16, 17, 27]. This technology enables therapists for the first time to measure the quantitative effects of HT for patients with DD in the early postoperative period. It has also been applied in a proof of principle study in our department and to reveal the physiological response to HT in healthy individuals [24]. Rendell et al. demonstrated this using laser doppler; they observed local hyperemia after focal exercise, which was independent of generalized hyperemia [14].

Although conditions in patients with DD might differ from healthy individuals, the most physiological circumstances and reactions were confirmed on the control digit. Therefore, the control digit served as an intraindividual benchmark, i.e., well perfused and not swollen (highest StO2, NIR THI and lowest TWI) before surgery. In addition, we observed differences preoperatively between primary and recurrent DD, significantly for NIR for all ROI being higher and THI for the AD being lower in primary DD.

In contrast the affected regions (palm and affected digit) showed significantly lower StO2 and NIR and higher TWI preoperatively: they were less perfused, less oxygenated and, in the sense of subtle inflammation or altered lymphatic drainage, swollen [4]. This is probably best explained by the microvascular alterations in the DD tissue itself, especially constrictive remodelling and a more constrictive muscular layer in the artery itself [26, 28].

After surgery before HT (OP + 1) we observed decreased oxygen supply and hematoma at the affected digit (reduced StO2 and NIR and simultaneously increased THI).

The palm showed hyperemia as a sign of possibly beginning inflammation (increase of THI and NIR) while being immobilized and compressed (no change in TWI). This is partially seen in the control digit (increased THI), although an increase in NIR could not be observed as in the palm. It remains questionable whether an increase of NIR can be expected at the fingertip in a depth of up to 6 mm, as here mainly solid structures such as bone and tendon are found. As no tissue is disturbed in the control digit, this is the only location where inflammatory processes, ergo edema (increased TWI), can expand in a closed space while the outflow is hindered by proximal compression in the palm. Here, the edema also does not compete with hematoma as in the palm or affected digit.

At OP + 1 the palm shows physiological behavior (reduced NIR and TWI, increased StO2 and THI) after HT, at the affected digit HT improves merely oxygenation and perfusion (increased StO2 and THI).

High oxygen saturation appears to have a positive impact on leucocyte function and collagen deposition, preventing wound infection and enhancing wound healing [29, 30]. The DD patients generally have restricted microcirculation because of occluded microvessels found in the immediate vicinity of DD tissue [28]. This might not only be one of the possible etiologies of DD itself but also the basis for wound healing disorders after partial fasciectomy [28, 31]. Another complication is the loss of range of motion achieved intraoperatively. Considering the results of this study, immediate postoperative HT for the hand is essential; however, this is often not feasible in an outpatient setting or cannot be performed on an outpatient basis due to pain, making inpatient HT for these patients necessary.

Therefore, prevention of edema is essential and does not rely solely on elevation but must be supported by external forces such as mechanical compression by a hand therapist [32, 33].

At OP + 2 a decrease in inflammation and hyperemia are observed at the control digit in comparison to OP + 1 (reduction of THI). Even if a severe edema was not seen, we observed a further effective reduction of TWI at all ROIs by HT at OP + 2.

These objectively observed effects of HT (better oxygenation and perfusion, and tissue water reduction) are in contrast to a study by Herweijer et al. [34], who did not show any effect of postoperative HT in patients with DD according to their referral criteria; however, the follow-up interval in their study was relatively long and there no data were available if HT might have accelerated postoperative recovery. In contrast to Herweijer et al. the postoperative observation time was relatively short in our study and we could not demonstrate normalization of HSI parameters of the treated finger compared to the control finger. In our opinion, this justifies continuing the therapy beyond the period of hospitalization.

Our study has a few limitations, which have to be mentioned. First, although DD is a common complaint, the number of patients included into the study is too small for a subgroup analysis for different clinical parameters or even primary and recurrent DD; however, this limitation is shared by other studies [34]. Second, our study was not a randomized controlled trial but a case control study. Given our results, withholding HT from patients would be unethical [8]. Third, the quantitative differences in HSI parameters were relatively small. Most observed differences were only in the single digit percentage range, even if these are highly significant.

Fourth, the follow-up period was short. The preoperative values were not regained after 2 days. Therefore, we are not able to define the interval of return to stable microcirculatory parameters.

Finally, we defined no clinical endpoint, thus we do not know whether HT has an impact on clinical outcome on partial fasciectomy.

Future studies should take these limitations into account and include more patients with both hands, add a control group, investigate for a longer observation period and address other clinical outcome parameters such as pain severity, swelling and range of motion.

In conclusion, HSI enables a quantitative, quick and noninvasive assessment of therapeutic effects in the postoperative healing process. In patients with DD after partial fasciectomy it demonstrated the effectiveness of HT and justifies continuing HT beyond hospitalization.

## Data Availability

All data present in the manuscript will be made available upon reasonable request.

## Abbreviations

DD: Dupuytren’s disease
HT: Hand therapy
HSI: Hyperspectral imaging
NIR: Near infrared perfusion index
StO2: Relative oxygen saturation
THI: Tissue hemoglobin index
TWI: Tissue water index

## Declarations

For this article no studies with human participants or animals were performed by any of the authors. All studies mentioned were in accordance with the ethical standards indicated in each case.
